# Hippocampal architecture viewed through the eyes of methodological development

**DOI:** 10.1007/s12565-025-00878-7

**Published:** 2025-08-05

**Authors:** Ling Zhao, Nicola Palomero-Gallagher

**Affiliations:** 1 Department of Psychology, School of Public Policy and Management, Nanchang University, Nanchang, 330000 China; 2 Institute of Neuroscience and Medicine (INM-1), Research Centre Jülich, 52425 Jülich, Germany; 3 C. & O. Vogt Institute for Brain Research, Heinrich-Heine-University, 40225 Dusseldorf, Germany

**Keywords:** Hippocampus, Structural organization, Methodological advances

## Abstract

This review focuses on the structural organization of the hippocampus and how our understanding of its cellular architecture and functional circuits has been enabled over the last 400 years by the development of methods as varied as the Golgi impregnation, immunohistochemical staining procedures, and polarized light imaging. We provide an outlook on how cutting-edge techniques such as high-resolution imaging and artificial intelligence may continue to shed light on the structural organization of the hippocampus and emphasize the importance of collaborative multidisciplinary efforts including classical neuroanatomists in this endeavor.

The hippocampus is one of the evolutionary oldest components of the cerebral cortex and among the first brain structures to be identified and described (Lewis 1923; Zilles 2004). It received its name in 1587, when the Italian anatomist Arantius wondered whether the macro-anatomy of this brain structure more closely resembled the shape of a silk worm or that of a small, upright swimming fish (Amaral and Lavenex 2007). This latter comparison resulted in the term hippocampus, derived from the Greek word *hippos* for “horse” and *kampos* for “sea monster”. Since then, our understanding of the structural organization of the hippocampus has developed over the centuries, driven by the insights provided by scientific methodological advancements ranging from classical histologic staining to modern molecular and genetic techniques. These technological advancements enabled researchers to revisit longstanding questions from fresh perspectives while also exploring entirely new ones (Morris et al. 2025). We here review several methods used in the study of the hippocampus and describe how they helped further our understanding of the architectonic organization of the hippocampus.

## Regions and layers of the hippocampus

The number of cytoarchitectonically distinct areas, which constitute the hippocampus, varies with different authors. According to the more conservative classification, the hippocampus, or hippocampus proper, is restricted to the Cornu Ammonis (CA) and the fascia dentata (FD), and the term hippocampal formation refers to the hippocampus and the subicular complex (DeFelipe et al. 2007; Duvernoy et al. 2005; Palomero-Gallagher et al. 2020; Rosene and Van Hoesen 1987; Witter 2012). More integrative neuroanatomists include the subicular complex in their definition of hippocampus, and consider the hippocampal formation to also encompass the entorhinal cortex (Amaral et al. 2007; Insausti and Amaral 2012). The present review will focus on the more conservative definition of the hippocampus proper and is thus restricted to the FD and CA regions. Furthermore, although the hippocampus exhibits highly conserved cytoarchitecture and connectivity across mammals, species differences do exist and will be mentioned where relevant.

**Fig. 1:**
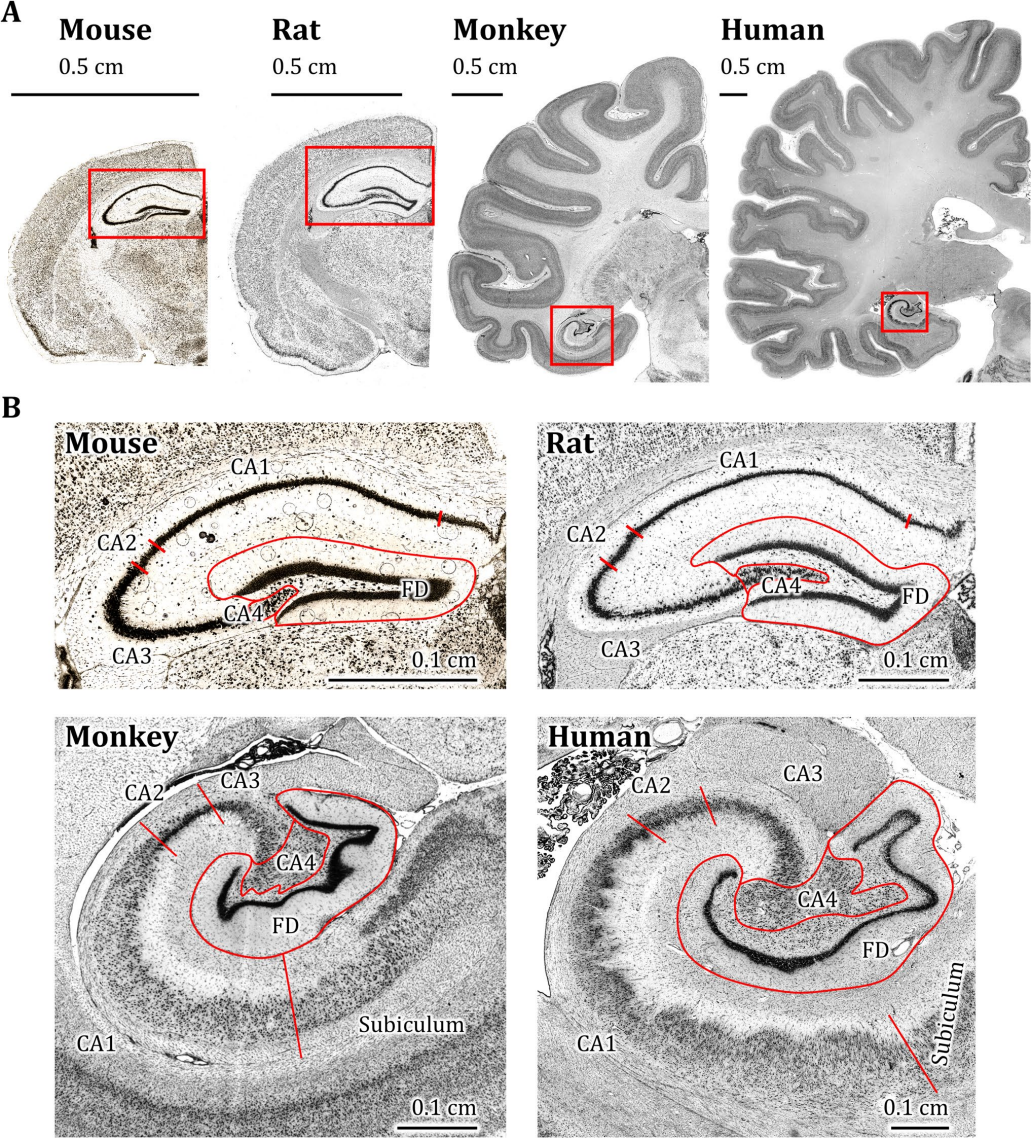
**A** Coronal sections of human, macaque monkey, rat, and mouse hemispheres stained for cell bodies, and in which the hippocampus is highlighted by red frames. **B** Detailed view of the hippocampus across these species, enabling comparison of species-specific differences in the thickness of the pyramidal layer relative to the total thickness of each Cornu Ammonis (CA) region. Note that the CA1 region is located dorsal to the fascia dentata (FD) in the mouse and rat brains, but ventral to the FD in the macaque and human brains. This flip in the relationship between the two regions is due to the change in the macroanatomic position of the hippocampus from beneath the corpus callosum to the medial part of the temporal lobe, and this change is driven by the expansion of the neocortex

Despite species-specific differences in the topological location of the hippocampus – it is found below the corpus callosum in primitive mammalians but is pushed ventrally and medially into the temporal lobe by neocortical expansion – the microstructure of these regions is remarkably conserved in eutherians, marsupials and monotremes, so that the distinct interleaved C-shaped configuration of the FD and CA regions can be easily identified across species (Fig. 1) (Insausti and Amaral 2012). The CA has been subdivided into the CA1-CA4 regions based, among other criteria, on differences in the packing density of its main neuronal type, the pyramidal neuron (Lorente de Nó, 1934; Palomero-Gallagher et al. 2020). Hereby, the CA4 region is surrounded by the concavity formed by FD, and the combination of these two cytoarchitectonically distinct entities builds the macroscopically identifiable dentate gyrus (Zilles et al. 2015). The existence of CA2 as a distinct hippocampal region has been the subject of some debate though, as discussed below, multiple structural characteristics warrant its classification as such (Ding and Van Hoesen 2015; Insausti et al. 2023; Oltmer et al. 2024; Palomero-Gallagher et al. 2020; Williams et al. 2023). The exact number of areas that can be defined within the subicular complex remains a subject of debate. Whereas some authors identify a prosubiculum (ProS), subiculum (or subiculum proper; Sub), presubiculum (PreS), parasubiculum (PaS) and transsubiculum (TrS), others consider ProS, PaS and TrS to be transitional areas rather than distinct architectonic entities (Ding et al. 2020; Ding 2013; Insausti and Amaral 2012; Palomero-Gallagher et al. 2020; Rose 1927; Rosenblum et al. 2024; von Economo and Koskinas 1925; Witter 2012; Witter and Amaral 2004).

**Fig. 2:**
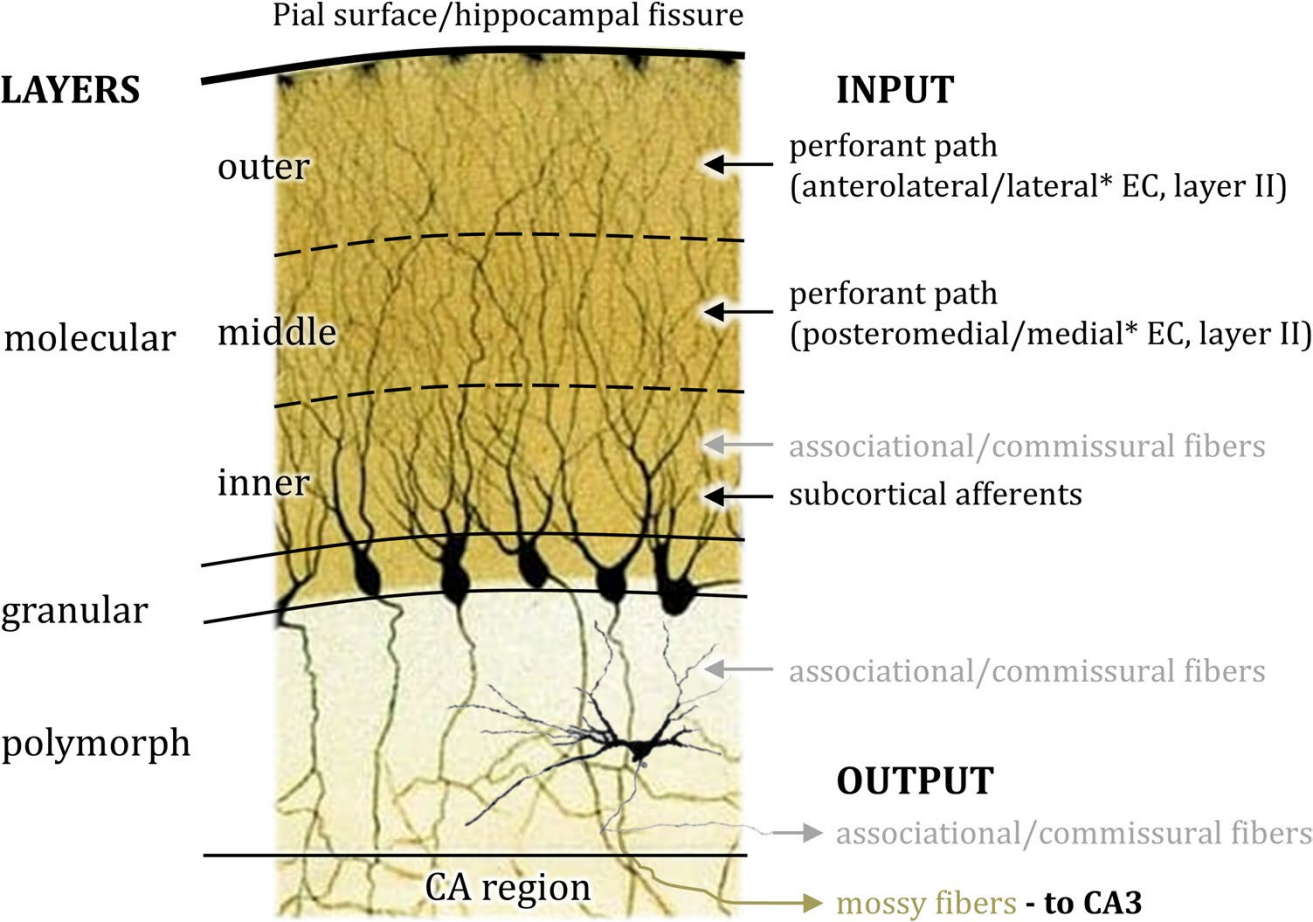
Layers of the fascia dentata and information concerning their input and output overlayed onto a modified drawing by Camillo Golgi (1885, part of Plate XXIII) depicting silver impregnated granule cells and onto which a drawing of a Golgi-impregnated mossy cell (after Amaral 1978, with permission) has been overlaid. Only the proximal axonal portion is depicted as more distal lengths are generally not identifiable in Golgi impregnations. * Note that associational and commissural fibers arise from the mossy cells located in the multiform layer of FD and target its ipsi- and contralateral molecular layer, respectively. Furthermore, the terms ‘anterolateral’ and ‘posteromedial’ refer to the macaque brain, whereas the terms ‘lateral’ and ‘medial’ refer to the rat brain. See the main text for further details. Also note that the granule cells and the mossy cell are not depicted to scale, as the soma of the latter is considerably larger than that of the former type (Amaral et al. 2025)

**Fig. 3:**
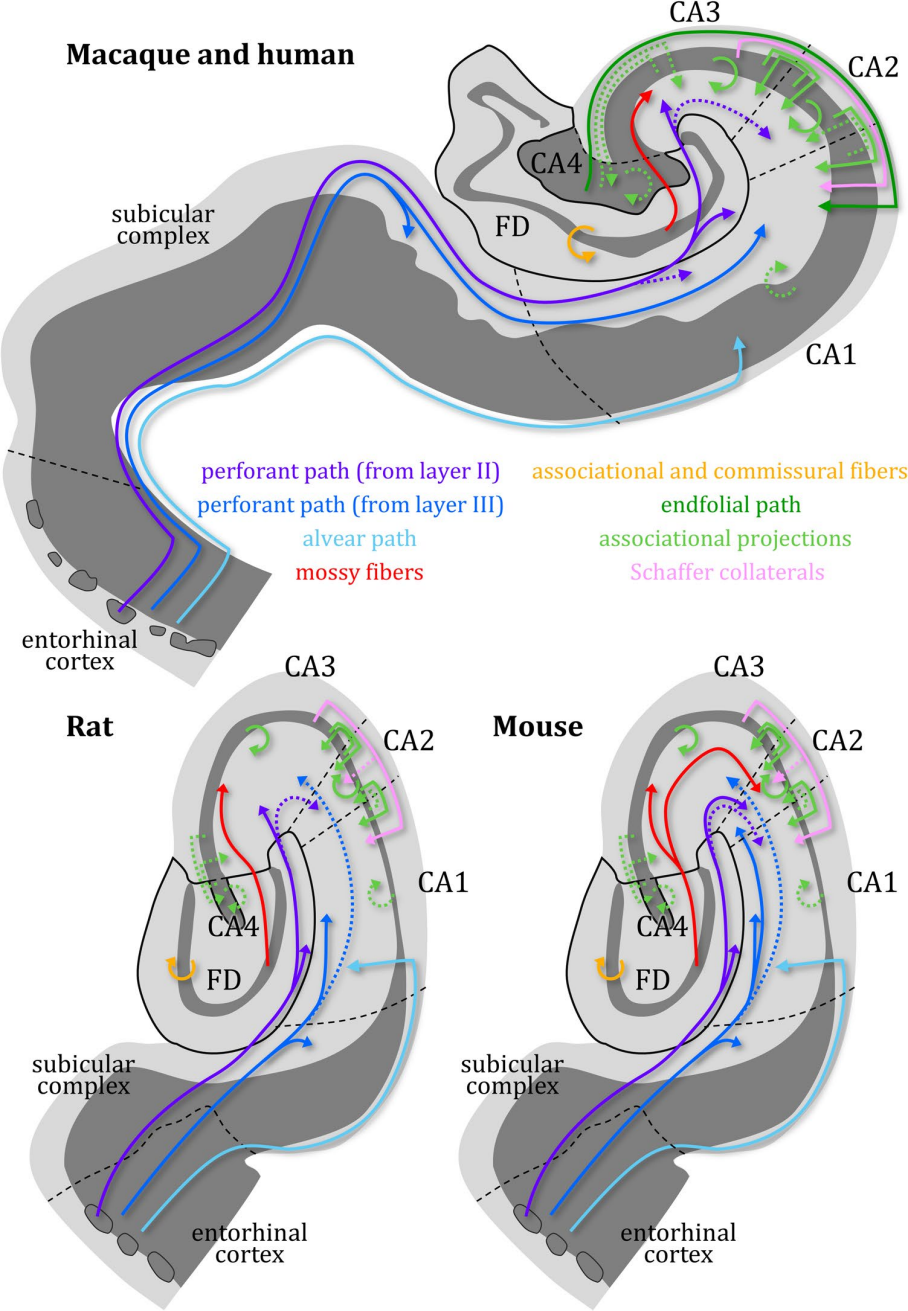
Hippocampal circuitry in the primate (human and macaque monkey), rat, and mouse brain. The drawings represent a coronally sectioned human and horizontally sectioned rat, and mouse hippocampi. Note that most of what we know about human hippocampal circuitry has been inferred from studies of the monkey brain, due to the limited direct access to human neural tissue. The alvear path is also known as the temporoammonic path. Projections from CA4 pyramids to the CA1 region form the endfolial path. Collaterals arising from CA3 pyramidal axons and targeting the CA1 region are called Schaffer collaterals. All other axon collaterals of pyramids located in the CA4-CA1 regions and targeting either themselves (e.g., from a CA3 pyramid to another CA3 pyramid either ipsi- or contralaterally) or any other CA region (e.g., from a CA3 pyramid to the CA4 or CA2 regions) are called associational projections. Dotted lines indicate only weak connectivity between the highlighted hippocampal regions

The hippocampus and areas ProS and Sub of the subicular complex belong to the archicortex and are thus per definition trilaminar, whereas PreS, PaS and TrS are periallocortical in nature (Stephan 1975). The **FD** presents a superficial cell-sparse layer followed by a very thin principal cell layer and deeper to that the polymorphic layer (Fig. 2):

- The molecular layer is occupied mainly by neuropil, though it also presents a small number of interneurons, most of which express the neuropeptide vasoactive intestinal peptide (VIP; Ribak and Seress 1983). The apical dendrites of granule cells span the entire width of this layer, and the deeper portion also contains dendrites of the pyramidal basket cells and of neurons from the polymorphic layer (Ribak and Seress 1983). Connectivity of the molecular layer also varies throughout its depth (Fig. 2). The outer two thirds are targeted mainly by layer II (and more weakly by layer V) of the entorhinal cortex via the perforant path (Amaral et al. 2014; Hevner and Kinney 1996; Kanter et al. 2025; Moser et al. 2017; Van Hoesen and Pandya 1975; Witter and Amaral 1991; Witter et al. 1989). Further, the outer and intermediate portions of the molecular layer receive input from different parts of the entorhinal cortex. In the macaque brain, the outer third of the molecular layer receives a stronger input from the rostral than from the caudal entorhinal cortex, and the opposite holds true for the middle molecular layer (Witter and Amaral 1991; Witter et al. 1989). In the rat, and mouse brain, the outer molecular layer is targeted by the lateral entorhinal cortex, whereas the middle layer receives input from the medial entorhinal cortex (van Groen et al. 2002, 2003; Witter 2007b). Finally, it should be noted that in non-human primates and rodents perforant path projections are also topographically organized along the longitudinal axis of the hippocampus, whereby the more lateral portions of the entorhinal cortex project to caudal levels of the molecular layer and its medial portions project to anterior levels of FD (Dolorfo and Amaral 1998; Kanter et al. 2025; van Groen et al. 2003; Witter and Amaral 1991; Witter et al. 1989), and this principle also seems to hold true for the human brain (Reznik et al. 2024). In addition to input via the perforant path, the outer third of the molecular layer receives a moderate but highly arborized serotonergic innervation(Amaral and Campbell 1986) and the inner third is targeted by subcortical structures such as cholinergic nuclei in the basal forebrain and GABAergic neurons from the supramammillary area (Amaral and Campbell 1986; Haglund et al. 1984; Mesulam et al. 1983; Nitsch and Leranth 1993, 1994; for a comprehensive review see Spruston et al. 2025). Finally, the inner third of the molecular layer also receives projections from the polymorphic layer of the ipsi- and contralateral FD via associational and commissural fibers, respectively (Amaral et al. 1984). See the section Invasive and non-invasive tract tracing methods for details of the method that led to these findings.
- The granular layer is composed mainly of the densely packed cell bodies of the granule cells, which constitute the FD’s principal cell type (Golgi 1885) and use glutamate as a transmitter (Clements et al. 1990; Crawford and Connor 1973; Storm-Mathisen et al. 1983). Isolated interneurons are also found within this layer and at its interface with the polymorphic layer (Ribak and Seress 1983). The pyramidal basket cells constitute the most notable example of this latter type of interneuron. The dendrites of granule cells are spiny and the apical tree branches repeatedly, forming a conical innervation domain within the molecular layer. Approximately 20% of granule cells in the adult human FD also display basal dendrites, as do about 9% in the macaque monkey brain (Seress and Mrzljak 1987). These dendrites are found mainly in the deep part of the granular layer, though a small portion reaches into the polymorphic layer or changes direction abruptly and extends into the deeper part of the molecular layer (Seress and Mrzljak 1987). In contrast, granule cells in the adult rodent brain do not display basal dendrites, though they are present during early developmental stages (Seress and Pokorny 1981). Axons of granule cells are not myelinated and were named mossy fibers (fibras *musgosas*) by (Ramón y Cajal 1893). They target the CA3 pyramids (Fig. 3), and along their course through the polymorphic layer also form synapses with mossy cells (see below Golgi impregnation). A study combining genetic manipulation (see below Genomic technology) and immunohistochemistry revealed that the mouse CA2 region is also targeted by granule cell axons along its entire longitudinal axis (Kohara et al. 2014; Llorens-Martín et al. 2015). However, in contrast to the mossy fiber contacts in the CA3 region (see below), pyramids in CA2 receive small mossy fiber boutons (Kohara et al. 2014).
- The polymorphic layer (or multiform layer) receives its name from the multiple cell types of which it is composed. The most frequent cell-type populating this layer is the mossy cell, a large multipolar glutamatergic neuron (Amaral 1978; Soriano and Frotscher 1994), although it also presents a myriad of different types of interneurons (Slomianka and Geneser 1993). See further below Golgi impregnation and Immunohistochemical stainings for more details concerning these GABAergic neurons. The polymorph layer receives a heavy noradrenergic innervation and, in a thin strip directly adjacent to the granular layer, is targeted by serotonergic terminals (Amaral and Campbell 1986; Azmitia and Segal 1978; Oleskevich et al. 1989). The polymorphic layer is often referred to as the hilus (Amaral et al. 2007; Insausti and Amaral 2012; Witter 2012), although this term has also been used at times to designate the combination of polymorphic layer and CA4 region(Braak et al. 1991; Frahm and Zilles 1994; Vogt and Vogt 1919; von Economo and Koskinas 1925).

The terminal portion of CA’s principal cellular layer encroaches into the concavity created by FD. Some authors identify this as a distinct area, namely **CA4** (also called CA3h) (Braak et al. 1991; Ding and Van Hoesen 2015; Frahm and Zilles 1994; Lim et al. 1997; Lorente de Nó, 1934; Palomero-Gallagher et al. 2020; Williams et al. 2023), whereas others believe it should be classified as being part of the CA3 region (Amaral and Insausti 1990; Amaral et al. 2007; Insausti and Amaral 2012; Witter 2012). CA4 consists of modified pyramids, which tend to form clusters and have lost their typical polarization into apical and basal dendritic trees, thus more closely resembling multipolar cells than CA3 pyramids and justifying the segregation of both areas (Braak et al. 1991; Palomero-Gallagher et al. 2020; Williams et al. 2023). Furthermore, differences in receptor architecture, which will be discussed in detail below (Receptor autoradiography) confirming the presence of a border between CA4 and CA3 (Palomero-Gallagher et al. 2020).

**Fig. 4:**
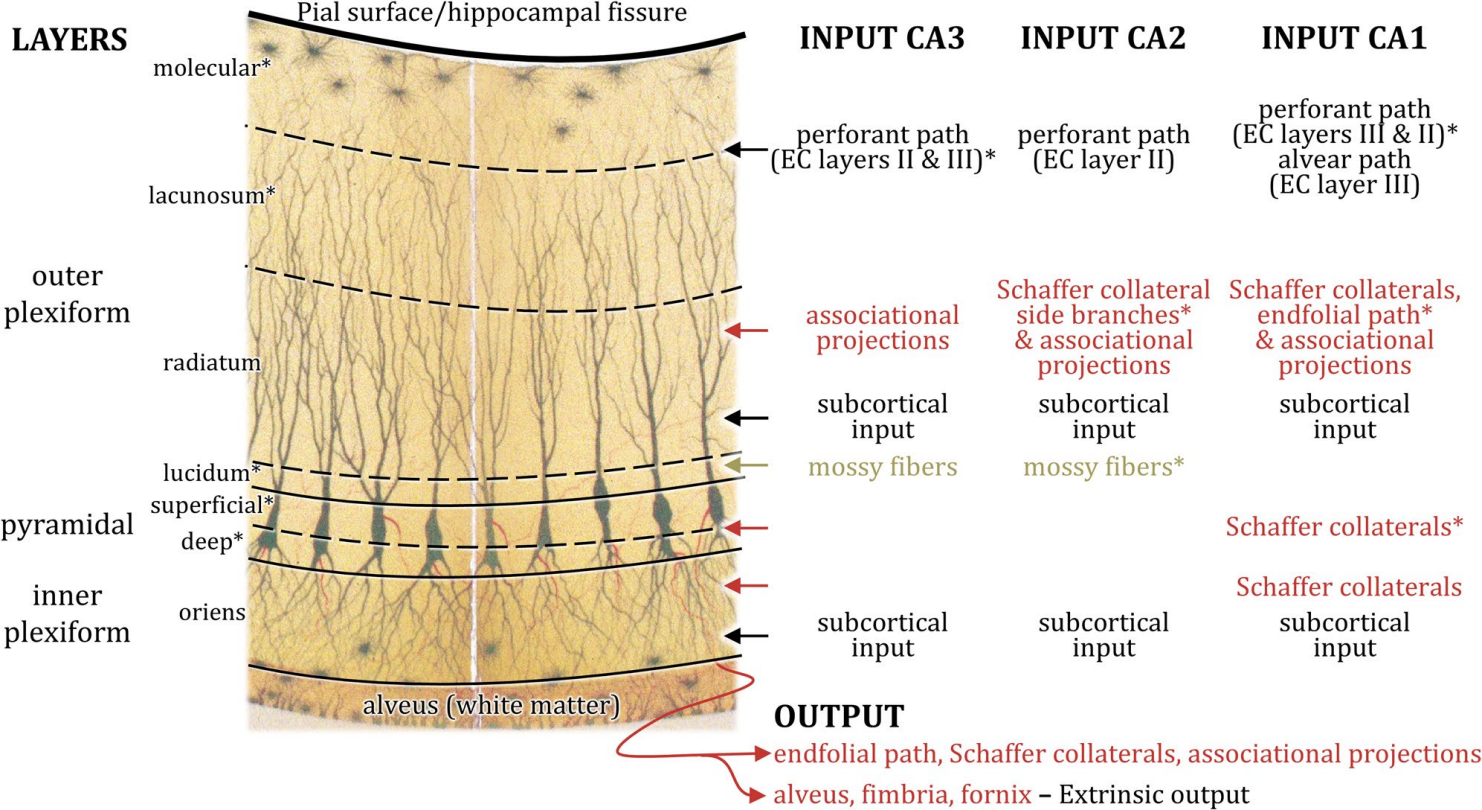
Layers of the CA region and information concerning their input and output overlayed onto a drawing by Camillo Golgi (1885, Plate XXI) depicting silver impregnated hippocampal pyramidal neurons. *Note that: 1) the lacunosum and molecular layers are often referred to jointly as a single laver (i.e., the lacunosum-molecular layer). 2) The lucidum layer is only present in the CA3 region. 3) The pyramidal layer is only subdivided into superficial and deep layers in CA1 and CA2. 4) Projections from layer III of the entorhinal cortex (EC) to the CA3 region and from layer II to the CA1 region have only been observed in the rat, and mouse brain, respectively (see text for details). 5) Side branches of the Schaffer collaterals innervating the CA2 region have only been observed in mice. 6) The endfolial path has only been observed in the human and macaque monkey brain. 7) Innervation of the CA2 region by mossy fibers has only been observed in mice. 8) Schaffer collaterals also target the pyramidal layer of the primate CA1 region, but not of the rodent CA1 region. Refer to the caption of Fig. 3 for information on the difference between Schaffer collaterals and associational projections. Only the proximal axonal portion is depicted, as more distal lengths are not identifiable

The **CA3-CA1** regions each present a so-called principal cellular layer flanked by cell-sparse plexiform layers, the outer of which has been divided into sublayers (Fig. 4). Differences in the packing density of the cellular layer and in the number of sublayers that can be identified within the outer plexiform layer constitute the criteria to delineate each of these three CA regions. Some authors include the hippocampal white matter in their definition of the inner plexiform layer. Six (sub)layers can be identified in CA3 when moving from the pial surface to the interface with the white matter: the molecular, lacunosum, radiatum and lucidum layers within the outer plexiform layer, and the pyramidal and oriens layers, which constitute the cellular and inner plexiform layers, respectively. CA2 and CA1 do not have a lucidum layer and thus each display five (sub)layers.

- The molecular layer is the outermost layer of the CA region and composed of neuropil and some scattered cells (Ramón y Cajal 1893).
- The lacunosum layer is also composed mainly of neuropil, but only presents a few isolated interneurons. In addition, it is rich in a plexus of horizontally arranged myelinated fibers with varied origins and targets (Ramón y Cajal 1893; Schaffer 1892).
  The lacunosum and molecular layers are mostly referred to collectively as the lacunosum-molecular layer due to their structural continuity and similarity, and to their shared functional role in synaptic connectivity and signal integration (Insausti and Amaral 2012; Lorente de Nó, 1934; Witter 2012). The lacunosum-molecular layer contains the distal portion of the apical dendrites of the CA pyramids and is targeted mainly by the perforant pathway (Fig. 4). Although the organization principles of most projections from the entorhinal cortex to the CA region are constant across species, there are some surprising differences, mainly concerning the CA3 and CA2 regions.
  In the CA3 region of monkeys and rats the lacunosum-molecular layer receives input from layer II neurons of the entorhinal cortex (Amaral et al. 2014; Ramón y Cajal 1909; Witter and Amaral 1991). In rats CA3 is also targeted by perforant path fibers originating in layer III of the entorhinal cortex, though this projection is not as dense as that arising from layer II (Witter 2007b). Interestingly, the CA3 region of the C57BL/6J mouse strain does not receive input from layer II of the entorhinal cortex, but is targeted by layer III neurons (van Groen et al. 2002, 2003), and further research is necessary to determine whether this applies to mice in general or is strain-specific (Witter 2007a).
  Projections arising from layer II of the entorhinal cortex and targeting CA2 have been described for monkey, cat and rat brains, though they are weaker than those reaching the CA3 region (Ino et al. 1998; Steward and Scoville 1976; Witter and Amaral 1991; Witter et al. 1989). In addition, in the rat brain input from the lateral entorhinal cortex was found to be stronger than from the medial entorhinal cortex (Lopez-Rojas et al. 2022; Masurkar et al. 2017). Concerning CA2 in the mouse brain, some researchers report that it receives input from both layer II and layer III entorhinal neurons (Chevaleyre and Siegelbaum 2010), whereas others report that this input arises solely from layer II (Kohara et al. 2014).
  Perforant path projections to the CA1 region of mammals originate mainly in layer III of the entorhinal cortex (Amaral et al. 2014; Ramón y Cajal 1909; Witter and Amaral 1991; for recent reviews see Amaral et al. 2025 and Kanter et al. 2025), though a very weak projection from layer II neurons has also been identified in the mouse brain (Kitamura et al. 2014; Ohara et al. 2019) (Fig. 3). Axons originating in layer II of the entorhinal cortex target the CA2 and CA3 regions following a laminar and topographical organization comparable to that described above for the FD. The lateral entorhinal cortex of the rat (anterolateral portion of the monkey) projects to the most superficial part of their lacunosum molecular layer, and the medial entorhinal cortex of the rat (posteromedial portion of the monkey) to its intermediate/deeper portion (van Groen et al. 2002; Witter 1989; Witter and Amaral 1991). Concerning layer III projections, those arising in the macaque anterolateral (rat lateral) and posteromedial (rat medial) parts of the entorhinal cortex target the portion of CA1 closest to the subiculum and to the CA2 region, respectively (Amaral et al. 2014; Witter and Amaral 1991; Witter et al. 1989). Interestingly this topographical organization of the perforant path to CA1 is mirrored by the projections from this region back to the entorhinal cortex (Witter and Amaral 2021). The lacunosum-molecular layer of CA1 also receives entorhinal projections via the alvear path (Deller et al. 1996; Ramón y Cajal 1909), serotonergic and noradrenergic input from the raphe nuclei (Azmitia and Segal 1978; McKenna and Vertes 2001) and the locus coeruleus (Oleskevich et al. 1989), respectively, and is strongly targeted by the nucleus reuniens (Vertes et al. 2006).
- The radiatum layer is also composed of neuropil and scattered interneurons (for details see Immunohistochemical stainings), though they are much more abundant than in the lacunosum-molecular layer. It contains the proximal portion of the apical dendrites of CA pyramidal neurons and is the target of both intrinsic and extrinsic projections.
  The *intrinsic* projections to the radiatum layer arise mainly from the axon collaterals of CA pyramids, which also target the pyramidal and oriens layers. In the rodent brain, the radiatum and oriens layers present comparable innervation densities via these collaterals, but the pyramidal layer is only very sparsely labeled (Hjorth-Simonsen 1973; Ma et al. 2006). In contrast, in macaques this sparse labeling of the pyramidal layer is restricted to the CA3 region (Kondo et al. 2009; Shinohara et al. 2012). The CA4-CA1 regions differ in the innervation pattern of their collaterals. CA4 axons mainly target CA1, and only a few terminate in CA3 or innervate other CA4 pyramids (Hjorth-Simonsen 1973; Ishizuka et al. 1990; Lim et al. 1997; Lorente de Nó, 1934; Zeineh et al. 2017). The projection from CA4 pyramids to the CA1 region, which has been described in humans and macaques, but not in rats or mice, has been called endfolial path (Lim et al. 1997; Zeineh et al. 2017). CA3 gives rise to extensive projections to itself and to the CA2 and CA1 regions (Hjorth-Simonsen 1973; Ishizuka et al. 1990; Kondo et al. 2009; Lorente de Nó, 1934; Ma et al. 2006; Rosene and Van Hoesen 1977). Collaterals of CA2 also project to other levels of CA2 and to CA1, and in addition project back to CA3 (Kondo et al. 2009). The CA1 region only gives rise to very weak projections to itself and to CA2, but never to CA3, since the main target of CA1 collaterals is the subiculum (Blatt and Rosene 1998; Kondo et al. 2009; Lorente de Nó, 1934). With the exception of those from CA3 to CA1, all these collaterals are collectively known as associational projections (Fig. 3) (Insausti and Amaral 2012; Lorente de Nó, 1934; Szirmai et al. 2012; Witter 2012). The collaterals of the giant CA3 pyramids which target the CA1 region have been named Schaffer collaterals after the first neuroanatomist to describe them (Lorente de Nó, 1934; Schaffer 1892). These collaterals form synapses on both pyramids and interneurons (Ma et al. 2006), and are particularly conspicuous because of their relatively large diameter. Schaffer collaterals can also be clearly identified by the fact that they reach up into the lacunosum layer, where they form a rich plexus of horizontally running myelinated fibers. In the mouse brain, Schaffer collaterals present side branches which also innervate CA2 pyramids (Kohara et al. 2014). In rats, associational projections terminate either ipsilaterally to the field of origin, or decussate through the hippocampal commissure to reach their respective targets in the contralateral hippocampus throughout the entire length of the hippocampus (Blackstad 1956; Cenquizca and Swanson 2007; Ishizuka et al. 1990; Ma et al. 2006; Witter 2012). In macaques, this decussation is only observed in the rostral portion of the hippocampus (Amaral et al. 1984). In the mouse brain, the radiatum layer of the CA2 region is also targeted by axons from granule cells of FD (Kohara et al. 2014).
  The radiatum layer receives *extrinsic* input from a variety of subcortical nuclei: CA3 and to a lesser extent CA2 and CA1 receive cholinergic input from the septal nuclei and the diagonal band of Broca (Ma et al. 2006; Schwegler et al. 1996). In rodents the deepest portion of the radiatum layer in CA3 and CA2, directly adjacent to the pyramidal layer receives serotonergic input from the raphe nuclei (Azmitia and Segal 1978; McKenna and Vertes 2001) and the locus coeruleus (Oleskevich et al. 1989). Although CA4 does not have a radiatum layer, it is also heavily targeted by the serotonergic and noradrenergic systems (Azmitia and Segal 1978; McKenna and Vertes 2001; Oleskevich et al. 1989; Powers et al. 1988).
- The lucidum layer is present only in the CA3 region. In cell body stainings it is visible as a cell-free strip between the radiatum and pyramidal layers and in sections processed with the Timm’s sulfide silver method it stands out as a darkly stained strip above and within the pyramidal layer (for more details see Functionally selective histologic stainings). It contains the axons of the granule cells (i.e., the mossy fibers) and is the site at which they form the *en passant* synapses on the proximal dendrites of CA3 pyramids (Amaral and Dent 1981; Lim et al. 1997).
  Connectivity between granule cells and pyramids is not reciprocal. I.e., The CA3 field does not project back to the FD (Hjorth-Simonsen 1973; Ishizuka et al. 1990; Kondo et al. 2009). Interestingly, however, a retrograde tracing study in the pilocarpine rat model demonstrated that CA3 pyramids of chronic epileptic rats do project back to the FD, where they specifically target the inner third of the molecular layer (Lehmann et al. 2001). This aberrant innervation pattern, together with connectivity anomalies within the CA region, was interpreted as subserving the hippocampal epileptic discharges (Lehmann et al. 2001).
- The pyramidal layer (the principal cell layer) is mainly composed of the cell bodies of the glutamatergic pyramidal neurons which constitute the principal cell type of the CA region (Lorente de Nó, 1934; Ramón y Cajal 1893; Somogyi et al. 1983). It also presents numerous kinds of interneurons that differ in their morphology, connectivity and physiologic properties (Lorente de Nó, 1934; Wheeler et al. 2024, 2015), and which will be discussed further below (Immunohistochemical stainings).
  Lorente de Nó (1934) divided the CA into four regions based mainly on differences in the morphology and packing density of their pyramidal neurons. See Golgi impregnation for details concerning Lorente de Nó’s detailed descriptions of these different CA-pyramids. In general terms, CA4, CA3 and CA2 contain larger pyramids than those found in CA1 (Insausti and Amaral 2012; Palomero-Gallagher et al. 2020; Witter 2012). Furthermore, CA3, CA2 and CA1 differ in their cell packing density, which in the primate brain also results in variations in the thickness of their pyramidal layer relative to their total cortical depth (Williams et al. 2023). Specifically, CA3 is characterized by a very high cell packing density, CA2 presents the narrowest and most densely packed pyramidal layer, and CA1 the broadest and most loosely packed pyramidal layer (Fig. 1). Finally, the pyramidal layer of CA2 and CA1 has been divided into superficial and deep portions. Lorente de Nó (1934) described a subdivision of the pyramidal layer of CA1 into two sublayers: a superficial sublayer with one or two rows of densely packed pyramids and a deeper one with several rows of less densely packed pyramids. He also mentioned that the deeper layer was more pronounced in humans and non-human primates than in what he called “lower mammals” such as the mouse, rabbit, dog, or cat brain. Despite these cross-species differences in the relative thickness and degree of sublamination of the pyramidal layer of CA1, subsequent studies have shown that in many species (including rats and mice) the pyramids found in these two sublayers have different developmental origins and can also be distinguished in the adult brain by their protein and gene expression levels (for a comprehensive review see Slomianka et al. 2011). There is also accumulating evidence from modern techniques including multiphoton glutamate uncaging or genetic manipulation, that the CA1 pyramidal layer can be clearly subdivided in rat, and mouse brains into distinct sublayers based on differences in connectivity patterns and susceptibility to pharmacologic modulation (Arszovszki et al. 2014; Lee et al. 2014; Maroso et al. 2016; Masurkar et al. 2017; Thome et al. 2014). Further studies will be necessary to understand the extent to which cross-species differences in the relative thickness of the pyramidal layer of CA1 are of functional relevance because pyramids in the superficial and deep layers are influenced by different types of interneurons, which modulate how this hippocampal region receives, integrates and transmits information and may thus support more complex memory and spatial processing processes.
- The oriens layer is relatively narrow and was described by Ramón y Cajal (1893) as the polymorphic layer of the CA region. It contains the basal dendrites and the axon of the pyramidal cells as well as a few scattered cells, most of which are interneurons (Lorente de Nó, 1934; Ramón y Cajal 1893) (see Immunohistochemical stainings).
  The oriens layer directly abuts the hippocampal white matter. On the ventricular surface of the hippocampus the white matter is visible as thin strip, the alveus layer, which is formed by the axons from pyramidal neurons. In their course from septal to caudal (in the primate brain), or from septal to temporal (in the rodent brain), these axons gather into an increasingly thickening fiber bundle clearly visible as a ridge on the hippocampal ventricular surface. This is the so-called fimbria. Once the hippocampal tail is reached, the fiber bundle is no longer in contact with the FD and CA regions and receives the name fornix. It connects the hippocampus with the septum and the hypothalamus (Insausti and Amaral 2012; Saunders and Aggleton 2007; Witter 2012). Thus, the alveus, fimbria and fornix all contain hippocampal efferent fibers, and only differ in their topologic relation to the FD and CA regions.

## How methodological advances help improve our understanding of the hippocampus

### The Golgi impregnation

The silver chromate structural staining technique developed by Camillo Golgi, and which he named the reazione nera (black reaction) but has since been named after him (Golgi 1873; for historical reviews see DeFelipe 2015, 2025 and Bentivoglio et al. 2019), led to a major break-through in histology. The importance of this methodological advance lies in the fact that it enabled for the first time the visualization of complete individual brain cells due to the sparsity of stained neurons in any given tissue sample. It comes, however, with the drawback that the Golgi impregnation occurs randomly, and is thus not reproducible. Despite this limitation, contemporaries of Golgi now had a method with which they could simultaneously study the exact appearance of the three parts of a neuron (i.e., the cell body, dendrites and axon). The Golgi method is primarily used for the analysis of dendritic architecture, as it clearly reveals the number of dendrites and the point(s) at which they exit the cell body, the complexity of their branching pattern, as well as their size and location in relation to those of the neuronal cell body. Visualization of the entire axon and of its collaterals is only possible in tissue from young specimens, and further hindered by the fact that following their course across sections is not always possible. Thus, modern tract-tracing or fluorescent labeling techniques (see below Invasive and non-invasive tract tracing methods) are currently generally preferred for the detailed analysis of axonal projections. Despite this limitation, the Golgi impregnation constituted a giant step forward because it enabled neuroscientists to identify and characterize different types of neurons. Furthermore, they could do this in 3D because of the small size of neurons relative to the thickness of the processed tissue sections, and the possibility offered by light microscopy to successively bring different depths of the section into sharp focus (the video accompanying the historical review by Bentivoglio et al. 2019 clearly demonstrates this micro-focusing process).

Ramón y Cajal was the first to recognize the enormous potential of this novel method for the advancement of neuroscience (DeFelipe 2015). He not only refined it (Ramón y Cajal and Azoulay 1894), but (more importantly) the insights he gained from his extensive studies using this method led him to postulate two fundamental organizational principles that have revolutionized our understanding of the brain and still hold true: the "Neuron Theory" and the "Law of Dynamic Polarization" (Ramón y Cajal 1899, 1933). His analyses of Golgi-impregnated cells in the avian cerebellum (Ramón y Cajal 1888) and in the rabbit hippocampus (Ramón y Cajal 1904) can be considered as cornerstone observations for his neuron and directionality of information flow theories, respectively.

**Fig. 5:**
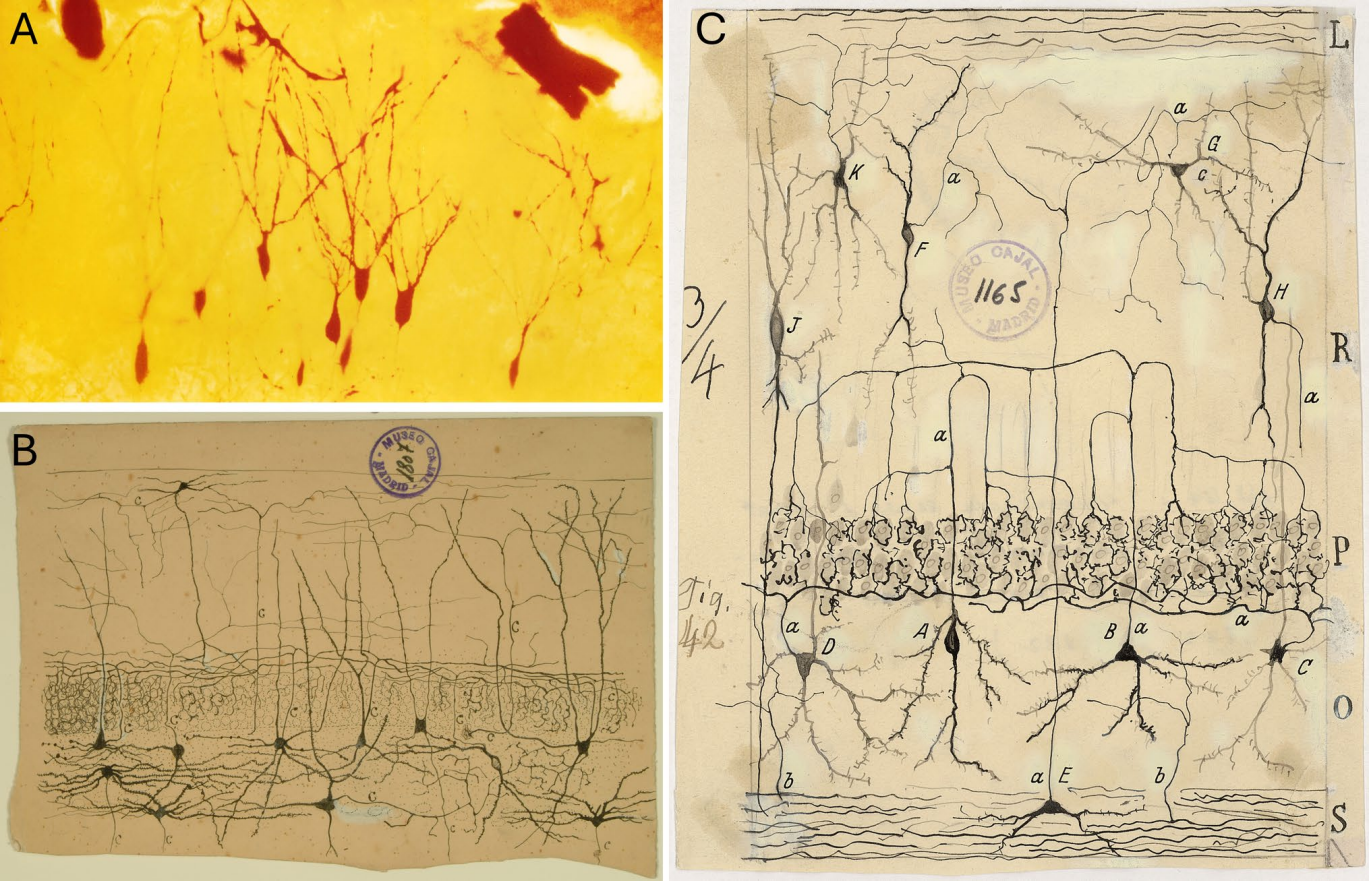
**A** Photograph of granule cells that Prof. Karl Zilles took of a Golgi-impregnation made by Ramón y Cajal. **B** Drawing by Ramón y Cajal of cells in the fascia dentata (FD). **C** Drawing by Ramón y Cajal of cells in the CA. Note that this drawing does not include the molecular layer or the superficial portion of the lacunosum layer. The beauty of his drawings not only reflect his skill in drawing, but are also particularly valuable because he conducted cutting-edge research and did not have access to microphotography or any other kind of imaging techniques. Thus, drawings (freehand or with the aid of a camera lucida) were the only method of depicting microscopic images. Photos in B and C: Legado Cajal (CSIC), with permission

Ramón y Cajal’s legacy also includes the first detailed description of the main neuronal types of the CA and FD regions, the pyramidal and granule cells, respectively (Ramón y Cajal 1893; Ramón y Cajal and Azoulay 1894). In his highly detailed drawings, Cajal depicts the location of the somata, dendrites and axons of these cell types relative to their laminar location (Fig. 5). He reported, e.g., that pyramids of the CA1 region have a much smaller cell body than that of CA2 or CA3 pyramids (Ramón y Cajal, 1893). Further, he described the prominent ascending collaterals of CA3 pyramids and the elaborate “thorny excrescences” in the initial portion of their apical dendrites (Ramón y Cajal 1893). These “thorny excrescences” are nothing other than the postsynaptic component of the mossy fiber synapse and are located in the lucidum layer of CA3.

Lorente de Nó (1934) continued and expanded on Ramón y Cajal (1893) studies using the Golgi impregnation. He provided detailed descriptions of the different types of CA pyramids found in each of its four divisions and characterized the relative location of synaptic terminals on CA3. CA4 is characterized by “modified pyramids” which more closely resemble multipolar neurons and thus lack a prominent apical dendrite (Lorente de Nó, 1934). The dendrites of these CA4 pyramids are covered in their entirety in spines, which are clearly larger in the proximal than in the distal portions of the dendritic tree. The larger, but not the smaller of these spines are the site of synaptic contacts with the mossy fibers (Lorente de Nó, 1934). Most of the axons of CA4 pyramids have a Schaffer collateral (i.e., a collateral which innervates CA1), but a few present only a short collateral which innervates either other cells within CA4 or reaches into the radiatum layer of CA3 (Fig. 3) (Lorente de Nó, 1934). Pyramids in CA3 are the largest of the CA region and their dendrites are covered in spines, whereby those in the initial portion of the apical dendrites (located in the lucidum layer) are conspicuously larger than the remaining ones. Their basal dendrites are particularly prominent, and their apical dendrites do not branch until they reach the radiatum layer. Lorente de Nó (1934) was the first to describe that although all CA3 pyramids have thick axons, not all of them have a Schaffer collateral, since some target CA2 or other cells within CA3 and thus represent associational projections. Pyramids in CA2 are almost the same as those of CA3, though with one important difference of functional relevance for hippocampal circuitry: although CA2 pyramids are comparable in shape and size to those of CA3, the initial portion of their dendritic tree is devoid of spines and is not targeted by the mossy fibers. The axon of CA2 pyramids has several collaterals, some of which are particularly long and terminate in the entorhinal cortex, whereas others form associational projections. Lorente de Nó (1934) stated that collaterals from CA2 pyramids do not target the CA1 region. However, Kondo et al. (2009) proved by means of a combined anterograde and retrograde study that CA2 does indeed project to CA1. CA1 is characterized by the smallest pyramids of the CA region and their dendrites are devoid of spines. The apical dendrites present numerous side branches which form a dense horizontal plexus within the radiatum layer.

In addition to the giant synapses on CA3 pyramids, mossy fibers innervate FD’s polymorph layer via a dense and complex pattern of collaterals. These collaterals establish so-called *en passant* synapses (though smaller than those with the CA3 pyramids) with mossy cells and with diverse types of interneurons (Acsády et al. [Bibr CR1][Bibr CR237]; Amaral 1979; Amaral and Dent 1981). As mentioned above, mossy cells are multipolar neurons with a highly branched dendritic tree and axon (Amaral 1978). They were named so because the large spines (resembling the thorny excrescences of CA3 pyramids) that cover the proximal portion of their dendrites gives them the appearance of being covered in moss (Amaral 1978). An elegant experimental approach combining the Golgi impregnation and an immunohistochemical staining confirmed that they use glutamate as a neurotransmitter and are thus excitatory in nature (Soriano and Frotscher 1994). The axon of mossy cells innervates the inner third of the ipsi- and contralateral molecular layer, and thus constitutes the hippocampal associational and commissural pathways (for reviews see Scharfman 2016, 2018). In addition to these projections, which can be both local and distant, mossy cells form synaptic contacts with the interneurons of the polymorph layer (Amaral 1978; Buckmaster et al. 1996; Frotscher et al. 1991).

The Norwegian neuroanatomist Theodore Blackstad also deserves a special mention in this section for his pioneering work in establishing methods that combine Golgi impregnation with electron microscopy and/or tract tracing techniques, thus greatly facilitating advances in the analysis of synaptic circuitry (for a historical review see Fairén, 2005). Of specific relevance for the present review, his research provided crucial insights into the regional and laminar organization of hippocampal commissural and associational fibers (Blackstad 1975; Blackstad et al. 1970; Blackstad and Kjaerheim 1961).

### Functionally selective histologic stainings

Functionally selective histologic staining techniques highlight specific cellular structures or biochemical activities based on their function rather than just their morphology. Although the usefulness of these techniques is limited by their sensitivity to peri-mortal environmental factors such as anesthesia or experimental conditions, in contrast to general stains such as the silver cell-body or myelin stainings (Gallyas 1979; Merker 1983), they provide valuable insights into metabolic states or neurotransmitter expression levels.

Developed in the late 1950s, the Timm stain is possibly one of the most frequently mentioned functionally selective histologic stainings in the framework of hippocampal research. It is based on the precipitation of zinc using silver sulfide (Timm 1958b), and selectively visualizes the chelatable zinc stored in synaptic vesicles in glutamatergic neurons (Ibata and Otsuka 1969). In the hippocampus the intense dark stain resulting from the precipitate highlights the trajectory of mossy fibers through the polymorph layer of the FD, the CA4 region and the pyramidal and lucidum layers of the CA3 region. This pattern was first described by the Timm himself in the rat and guinea hippocampus (Timm 1958a) and consistently replicated by numerous authors in other species, including macaque monkeys and humans (Insausti and Amaral 2012; Witter 2012). In addition, that the CA1 and CA2 regions are also characterized by a conspicuously higher concentration of zinc than that of the neocortex or adjacent mesocortical areas (Ichinohe and Rockland 2005), thus highlighting the importance of this trace element in hippocampal neurotransmission.

Histochemical stainings have also been used to characterize the aminergic innervation of the hippocampus and thus provide insights into control of its activity levels by modulatory neurotransmitters. A study combining retrograde tracing and staining for tyrosine hydroxylase (TH) revealed that dopaminergic mesencephalic nuclei target the hippocampus only very sparsely (Gasbarri et al. 1996, 1994). However, see further below (Genomic technology) for evidence that the locus coeruleus is also a source of dopaminergic projections to the hippocampus.

The acetylcholinesterase (AChE) and choline acetyl transferase (ChAT) stains visualize the enzymes responsible for the break-down and the synthesis of acetylcholine, respectively. Thus, ChAT is used to label the soma of cholinergic neurons whereas AChE can be used to identify regions targeted by their axons. AChE staining in the human hippocampus is conspicuously stronger in CA2-CA4 than in CA1, and more prominent in the cellular than the plexiform layers of CA1-CA3 (Green and Mesulam 1988), whereas in rodents it is stronger in the plexiform layers, particularly the lucidum layer (Slomianka and Geneser 1991, 1993). Furthermore, whereas in humans AChE staining intensity of the molecular and polymorph layers of FD is comparable to that of the pyramidal layer in CA1 and of CA4, respectively, the mouse molecular shows only very low levels of AChE activity (Green and Mesulam 1988; Slomianka and Geneser 1991, 1993).

### Immunohistochemical stainings

In contrast to functionally selective histologic staining techniques, immunohistochemistry is used to identify *single specific proteins* in tissue sections by exploiting the principle of antigen–antibody binding. In addition, this method enables the localization and relative quantification of protein expression levels with a high degree of spatial resolution. Ramón y Cajal’s legacy demonstrates that many organizational principles of the brain can be inferred by the analysis of the morphology of its cells. However, differences in shape and size are not the only things that count in life, and the advent of immunohistochemistry enabled scientists to determine the type of neurotransmitter released by each of these morphologically distinct neurons. Given that binding of different neurotransmitters to their receptors has different effects on activity levels of the target cell, this aspect of brain organization is particularly relevant to understand the emergence and modulation of networks subserving brain function (Palomero-Gallagher and Zilles 2018).

In February 1983, Storm-Mathisen et al. (1983) published the first selective immunohistochemical visualization of glutamate and GABA distribution patterns. This led to a breakthrough in hippocampal research because, as we now know, hippocampal neurons use either one of these two classical neurotransmitters for signal transduction. Further, they postulated that glutamate- and GABA-immunoreactive neurons were what at the time were considered excitatory and inhibitory neurons, respectively (Storm-Mathisen et al. 1983). In July of the same year, Somogyi et al. (1983) provided evidence that the principal cell type of the CA region, the pyramidal neuron, is indeed glutamatergic in nature. Interneurons can also be identified by visualization of glutamic acid decarboxylase, the enzyme which metabolizes GABA from glutamate (Ribak 1978).

**Fig. 6:**
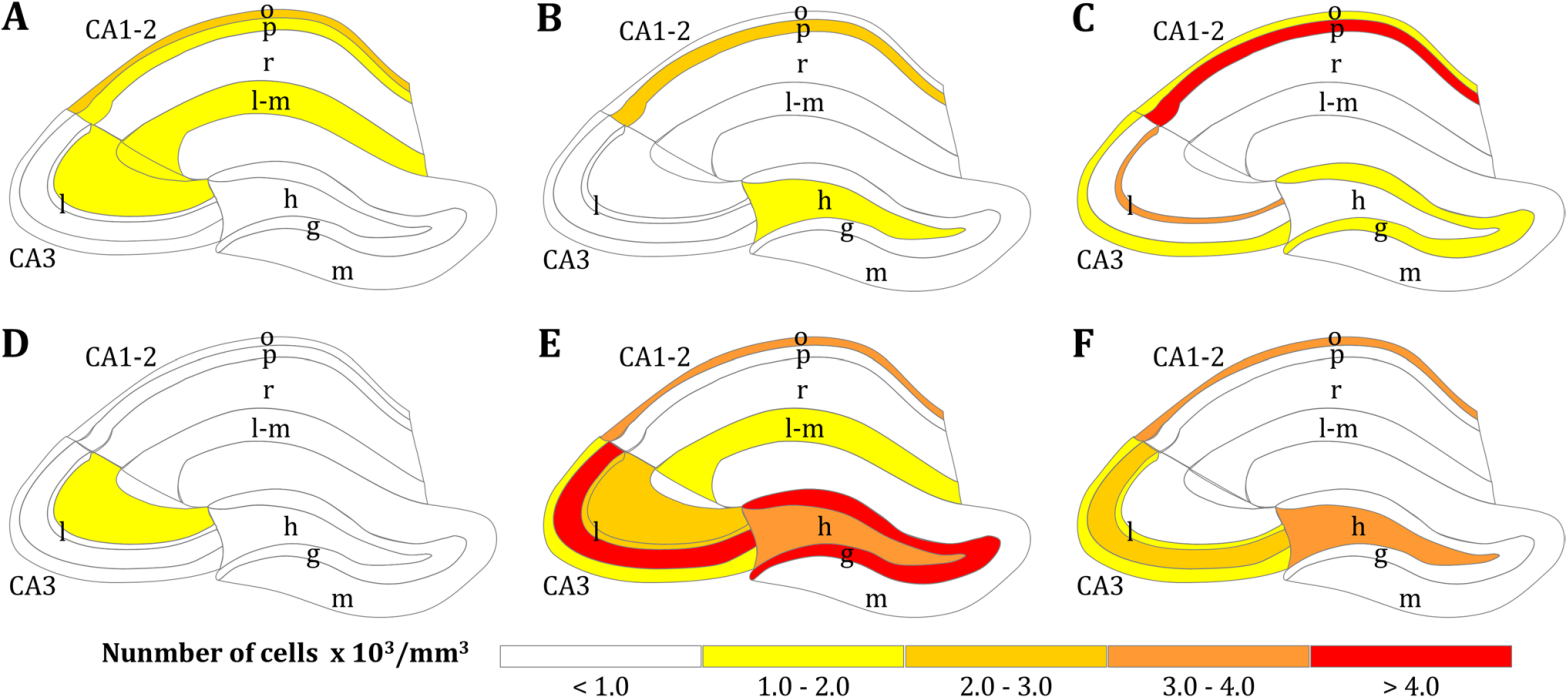
Interneurons expressing specific molecular protein markers identified by immunohistochemistry are differentially distributed across hippocampal regions and layers. Color coding indicates numerical density (in number of thousand cells per mm^3^) of interneurons expressing the calcium binding proteins calbindin (**A**), calretinin (**B**), and parvalbumin (**C**), or the modulatory neuropeptides cholecystokinin (**D**), neuropeptide Y (**E**), and somatostatin (**F**). Data taken from Jinno et al. (1998)

It does not suffice, however, to simply identify a GABAergic cell as such, because there are numerous types of interneurons. Although a detailed description is out of the scope of this review, it must be noted that interneurons not only differ in the shape and size of their dendrites and axons, or in the specific subcellular domain of pyramidal cells with which they establish synaptic contacts, but also in their firing activity. Thus, they can be classified into categories such as fast-spiking, burst-spiking or late-spiking interneurons based on their intrinsic firing patterns (for comprehensive reviews see Booker and Vida 2018; DeFelipe et al. 2013; Freund and Buzsáki 1996; Spruston et al. 2025; Tzilivaki et al. 2023; Wheeler et al. 2024). Through this morphologic, neurochemical and physiologic variety, different types of interneurons can differentially modulate neuronal microcircuits. The advent of immunohistochemistry meant that interneuron subtypes could also be identified according to their expressing a specific molecular marker or a combination thereof (Kepecs and Fishell 2014). These major markers are proteins related to GABA-mediated signaling and include compounds as varied as calcium binding proteins (calbindin, calretinin, parvalbumin) or proteins modulating synaptic strength (reelin, Purkinje-cell protein 4, chromogranin A), as well as modulatory neuropeptides co-released with GABA by some neurons (cholecystokinin, neuropeptide Y, somatostatin, vasoactive intestinal peptide), or enzymes which produce signaling molecules co-released with GABA (neuronal nitric oxide synthase). Interneurons expressing one or more of these markers are differentially distributed within the hippocampal regions and layers (Fig. 6) and have also been found to target distinct portions of the granular or pyramidal cells (Jinno and Kosaka 2006; Pelkey et al. 2017; Wheeler et al. 2024, 2015).

Immunohistochemistry can also be applied to visualize cellular components such as synaptic vesicles or the neurofilament proteins which compose the cytoskeleton of neurons. A study using SMI-31 and SMI-32, antibodies which specifically label the phosphorylated and non-phosphorylated epitopes of neurofilament H, respectively, revealed their segregated expression in the rat hippocampus (Mikuni et al. 1998). Whereas SMI-31-immunoreactivity was revealed in the mossy fiber pathway, thus highlighting the axons of FD granule cells, SMI-32 labeled neurons were mainly only present in the pyramidal cells of the CA region (Mikuni et al. 1998). Further studies have confirmed this selectivity and demonstrated that the lowest degree of SMI-32 immunoreactivity is found in pyramids of the CA2 region (Ding and Van Hoesen 2015; Lavenex et al. 2004; Morrison et al. 1987; Thangavel et al. 2009). The position and extent of CA2 are also highlighted by differences in the distribution of chromogranin A- and of Regulator of G-protein signaling-14-immunoreactivity (González-Arnay et al. 2024). Regulator of G-protein signaling-14 modulates downstream processes associated with activation of GTPase enzymatic activity (Traver et al. 2000), and chromogranin A is a protein released with neurotransmitters such as dopamine or serotonin which plays an important role in the formation of vesicles and the regulation of the secretion process via binding of Ca^2+^ (Dominguez et al. 2018; Smith 1971; Videen et al. 1992). Immunoreactivity for these two modulators of synaptic transmission was found to be stronger in the cell body of CA2 pyramids than in that of CA3 or CA1 pyramids (González-Arnay et al. 2024).

### Receptor autoradiography

Neurotransmitter receptors are proteins or protein complexes that are embedded in the cellular membrane and can bind to the chemical messengers released by neurons (i.e., neurotransmitters). Thus, they are key molecules in signal transmission and their heterogeneous distribution in the brain reveals the relationship between its structural segregation and functional organization principles (Palomero-Gallagher and Zilles 2018; Zachlod et al. 2023; Zilles et al. 2002). The regional differences in receptor distribution and density can be visualized and analyzed by means of quantitative in vitro receptor autoradiography, which utilizes radioactively labeled ligands that specifically bind to target receptors, followed by exposure to radiation-sensitive films or detectors to produce images of different receptor distributions (Palomero-Gallagher and Zilles 2018; Zilles et al. 2002). This method has the enormous advantage over immunohistochemistry, which only enables the visualization of individual proteins, in that it reveals the protein complexes embedded in the cellular membrane and in their native configuration. This strength, however, comes with the drawback of a lower spatial resolution than that provided by light or fluorescence microscopy techniques. It has the further advantage of revealing the regional and laminar distribution patterns of multiple receptors within the same brain sample and with a high resolution and of being fully quantifiable (Palomero-Gallagher and Zilles 2018; Zilles et al. 2002). Finally, the organization principles revealed by the simultaneous analysis of multiple receptor types in an architectonically identified brain region have been shown to be conserved throughout mammalian brains (Zilles and Palomero-Gallagher 2017).

Receptor autoradiography has been applied in multiple studies involving the rodent, non-human primate and human hippocampus (Biegon et al. 1982; Blatt et al. 2001; Castelli et al. 2000; Kraemer et al. 1995; Lothmann et al. 2021; Palomero-Gallagher et al. 2020; Zhao et al. 2023, 2025). The lucidum layer is clearly revealed by the kainate receptor, which presents significantly higher densities of this receptor type than do neighboring layers (Lothmann et al. 2021; Palomero-Gallagher et al. 2020). Thus, this finding emphasizes the importance of this glutamatergic receptor type in the transfer of information between the granule cells and the CA3 pyramids. In addition, for other receptors (e.g., AMPA, α_2_), differing density expressions in the inner and outer portions of the molecular layer of FD highlight how input from the medial and lateral parts of the entorhinal cortex is subjected to a different neurochemical regulation (Palomero-Gallagher et al. 2020). Within the CA layers, although the lacunosum and molecular layers are mostly merged into a single one based on cytoarchitecture, they can be distinguished by the higher densities of NMDA, α_2_, M_3_ and 5-HT_2_ receptors in the molecular than in the lacunosum component (Palomero-Gallagher et al. 2020). It is noteworthy that receptor autoradiography can also be used to reveal abnormal receptor expressions associated with neurologic and psychiatric disorders such as epilepsy and Alzheimer’s disease (Blatt et al. 2001; Graebenitz et al. 2011; Hand et al. 1997; Palomero-Gallagher et al. 2012; Westlake et al. 1994). Blatt et al. (2001) demonstrated that the GABA_A_ receptor exhibits significantly lower expression in the pyramidal layer of CA1 in autism patients compared with controls, a pattern also observed for GABA_A_/BZ binding sites in the pyramidal layer of CA2, indicating the association of the disease with a disturbed GABAergic neurotransmission.

**Fig. 7:**
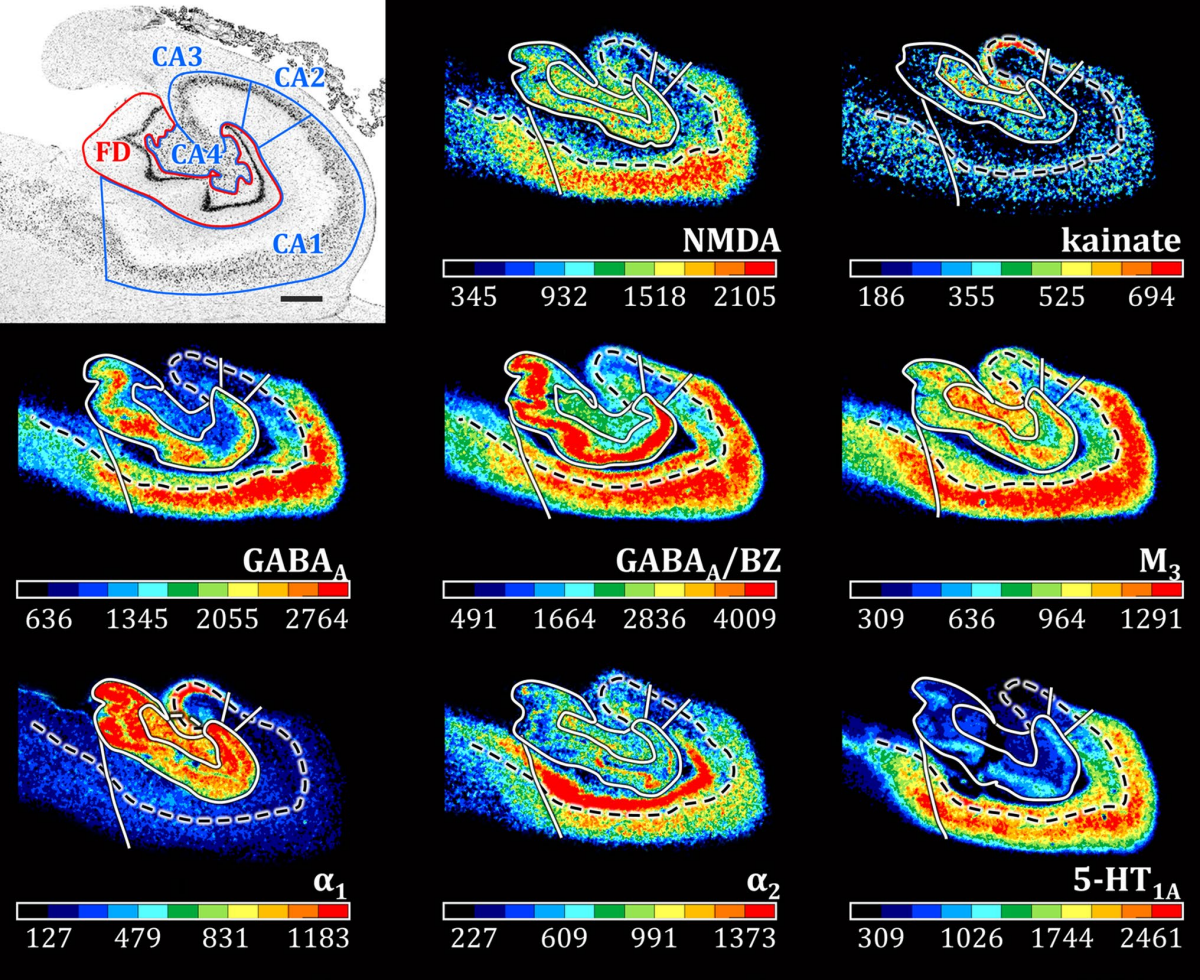
Cyto-and receptor architecture of the human hippocampus. The figure displays exemplary receptor autoradiographs through the body of a coronally sectioned human hippocampus (modified from Palomero-Gallagher et al. 2020, and clearly reveals the existence of distinct regional and laminar differences in molecular architecture

A recent combined cyto- and receptor architectonic analysis provides a comprehensive description of the regional and laminar distribution of 15 neurotransmitter receptors in the human hippocampal complex, which further validates the identification of CA4 and CA2 as distinct regions (Fig. 7) (Palomero-Gallagher et al. 2020). The border between CA2 and CA3 is clearly identifiable due to the conspicuously high kainate and α_1_ receptor densities in the lucidum layer (Palomero-Gallagher et al. 2020), which is specific of CA3 (Insausti and Amaral 2012; Witter 2012). The border between CA2 and CA1 is highlighted, e.g., by differences in the densities of GABA_A_, M_3_, α_2_ and 5-HT_1A_ receptors, as well as of GABA_A_/BZ binding sites, all of which are lower in CA2 than in CA1. The higher densities of NMDA, kainate and M_3_ receptors and of GABA_A_/BZ binding sites in CA4 than in CA3 support the definition of the former as a distinct region. Differences in the densities of, e.g., kainate, M_3_ or α_1_ receptors also highlight the border between CA4 and the polymorphic layer of FD.

Although in vitro receptor autoradiography offers unique advantages such as providing quantitative data at the microcircuit level which can be used for diverse modeling approaches (Froudist-Walsh et al. 2021; Klatzmann et al. 2025), the availability of high-affinity radioligands specific for a single receptor (sub)type remains an important bottleneck. A crucial development would be improving the spatial resolution to the single-cell level, as this would allow researchers to determine whether the labeled receptors are pre- or postsynaptic. The ability to visualize more than one receptor type per section, combined with single-cell resolution, would enable the analysis of distinct receptor expression patterns across different cell types.

### Genomic technology

Genomic technology enables analysis of the contribution of genetic factors to brain structure, function, and disease at various levels of complexity (Cembrowski et al. 2016; Chen et al. 2020; Zeisel et al. 2015; Zhou et al. 2024). The in situ hybridization technique, which was developed in the 1960s (Gall 2016), combines molecular biologic techniques with histologic and cytologic analysis of gene expression. Thus, it enables the localization of specific nucleic acid sequences within tissue sections, providing a highly resolved spatial context, though only in up to three different genes per section. In contrast, bulk sequencing techniques, including Sanger sequencing (Sanger et al. 1977) and next-generation sequencing (Goodwin et al. 2016), revolutionized genomic technology by enabling high-throughput sequencing of pooled cells, though these methods do not capture spatial information or cell-type specificity. These limitations were partially overcome with the advent of single-cell sequencing (Shapiro et al. 2013), which enables the analysis of individual cells, capturing their distinct genetic and transcriptomic profiles. However, since the method requires tissue dissociation, it provides no information concerning how these different cell types relate to each other in the brain. To bridge this gap, Ståhl et al. (2016) developed a new method which they called “Spatial Transcriptomics”, and which preserves tissue architecture while enabling transcriptome-wide profiling.

In situ hybridization has been applied to the human and mouse brains, providing high-resolution insights into their genomic organization and preserving architecture (Lein et al. 2007; Shah et al. 2016; Shen et al. 2012; Yao et al. 2023). In addition, the Allen Developing Mouse Brain Atlas (https://developingmouse.brain-map.org/) provides a comprehensive resource mapping gene expression throughout mouse brain development, which features high-resolution in situ hybridization image data across different prenatal and postnatal timepoints, revealing dynamic spatiotemporal gene expression in the hippocampus during brain development. These freely accessible resources have prompted numerous studies that have helped deepen our understanding of the genomic organization of the hippocampus. Such studies have revealed, e.g., the unique gene expression profiles of hippocampal regions, including CA2, and which can help predict functional differentiation across their longitudinal axis (Dudek et al. 2016; Lein et al. 2007; Thompson et al. 2008).

The field of genetic manipulation has also helped further our understanding of hippocampal structure–function relationships. Studies using Th-Cre and (DAT)-IRES-Cre transgenic mice revealed that not only the ventral tegmental area, but also the locus coeruleus provides dopaminergic innervation to the hippocampus (Kempadoo et al. 2016; Takeuchi et al. 2016). Furthermore, whereas dopaminergic innervation from the locus coeruleus is homogeneously distributed throughout the rat hippocampus, the oriens and pyramidal layers of the CA2 region are the most heavily targeted structures by the ventral tegmental area (Takeuchi et al. 2016). Combining the use of transgenic mice with optogenetic manipulation and electrophysiological recordings to shed light on the connectivity pattern of FD’s granule cells and their plasticity during learning and memory processes, Kohara et al. (2014) confirmed that granule cells not only project to the lucidum layer of CA3, but also to the deep portion of the radiatum layer in the CA2 region. Further, they demonstrated that optogenetic stimulation of mossy fibers induced large excitatory postsynaptic currents in both interneurons and pyramidal cells of the CA2 region (Kohara et al. 2014).

In recent years, spatial transcriptomics has emerged as a uniquely powerful tool to study the spatial gene-expression features of the hippocampus (Thompson et al. 2024; Vanrobaeys et al. 2023). It has the added value of also offering promising insights into neurologic disorders (Simard et al. 2024; Wang et al. 2025a, 2025b). Thompson et al. (2024) integrated spatially resolved transcriptomics and single-nucleus RNA-sequencing to construct a comprehensive molecular atlas of the adult human anterior hippocampus, capturing cell-type-specific profiles and spatial features. This open-access multimodal dataset provides a unique biologic perspective on the molecular neuroanatomy of the human hippocampus. Wang et al. (2025b) employed Stereo-seq spatial transcriptomic and single-nucleus RNA sequencing combined with immunohistochemistry and cell segmentation algorithms, to achieve precise spatial localization and typing of individual cells in the human hippocampus both with and without Alzheimer’s disease. Their study revealed a significantly decrease neuronal density in the CA1 region but not in CA4 of patients with Alzheimer’s disease, offering new insights into the disease’s molecular mechanisms. This differential vulnerability may be attributed to gene alterations in CA4 that confer resilience to AD-related neurodegeneration, and thus reveals potential avenues for precise diagnosis.

Despite the impact of spatial transcriptomics, several limitations remain, including insufficient resolution, low sensitivity for detecting rare transcripts, and computational challenges (e.g., deconvolving mixed signals) associated with data analysis. Moreover, it cannot directly capture dynamic processes, such as real-time transcriptional changes occurring during learning and memory. These limitations highlight the need for improvements in cellular resolution, multi-omics and cross-scale integration (e.g., spatial epigenomics, proteomics, fMRI or electrophysiology), and live-cell dynamic tracking. Such advancements could further uncover spatially defined molecular mechanisms underlying hippocampal function and provide novel insights into neurologic and psychiatric disorders.

At the interface between the fields of genomics and proteomics, the complex relationship between receptor densities and their corresponding encoding genes, as well as how these relationships change throughout hippocampal development, remains unclear. Zhao et al. (2023) studied the relationship between receptors for the classical neurotransmitters glutamate, GABA, acetylcholine, noradrenaline, serotonin and dopamine and their corresponding genes in the human hippocampus by combining the receptor autoradiography and bulk sequencing techniques. The widely varying correlation coefficients suggest that receptor expression in the human hippocampus is not controlled only by the corresponding RNA levels, but also by multiple regionally specific post-translational factors. Moreover, Zhao et al. (2025) characterized the distribution patterns of 18 neurotransmitter receptor types in the mouse hippocampus at P7 and compared it with the expression of the corresponding encoding genes at P4 in in situ hybridization images and found that the distribution of most analyzed receptors aligned with the expression of their primary encoding genes. Given the mismatch between gene expression levels and receptor densities, it is crucial to advance our understanding of the mechanisms underlying translation and receptor expression in the hippocampus. These processes involve multiple key steps, including correct protein folding and co-assembly in the endoplasmic reticulum, post-translational modifications, and subsequent trafficking to the appropriate membrane surface. For example, since ionotropic receptors are protein complexes, their functionality depends on the correct assembly of subunits to form an active receptor. For a comprehensive review of these processes, see Schwappach (2008) and Stephenson et al. (2008).

### Invasive and non-invasive tract tracing methods

Tract tracing methods serve to map the anatomic connections between neurons and brain regions, thus shedding light on how specific pathways support behavior, cognition, and sensory processing. Invasive approaches rely on the use of chemical or viral tracers in animal models and traditionally provide the gold standard for the analysis of connectivity patterns between neuronal populations. The advent of magnetic resonance imaging (MRI) brought the possibility of using non-invasive techniques to identify large-scale connectivity maps in living subjects. Invasive methods can be used not only to explore aspects of brain organization that cannot be studied directly in the human brain, but also to validate and refine the interpretations of non-invasive data, while non-invasive methods are crucial to extend anatomic insights to human studies, allowing for translational research and clinical applications.

Invasive tract tracing methods initially involved a localized cortical lesion and visualization with a metallic silver impregnation of the resulting fiber degeneration (for comprehensive reviews see Morecraft et al. 2014; Saleeba et al. 2019, 2020; Lanciego and Wouterlood 2020; Wang et al. 2023; Xu et al. 2020). This method gave way in the late 1960s to an approach which relies on the in vivo uptake of a tracer substance injected into the brain of an experimental animal and its transport to other regions via the corresponding interconnecting axons, followed by the ex vivo histologic processing of the brain for axonal visualization. Retrograde tracers are transported from the site of application to the cell body and serve to visualize the input received by a brain area, whereas anterograde tracers are transported to the synaptic terminals and reveal a brain region’s output. Tracers can be classified into two major groups depending on whether they are used to detect direct connections between two neuronal structures (i.e., non-transsynaptic tracers), or serve to analyze intercellular connectivity (i.e., the transsynaptic tracers and tracers passing gap junctions). Anterograde and retrograde tracing techniques are complementary in that the former provide detailed information concerning synaptic targets but may be less effective at labeling sparse or long-range projections, whereas retrograde tracers can produce signal that is useful for the identification of a broad set of connections, but do not necessarily resolve the details of afferent terminal organization.

Polarized light imaging (PLI) is a microscopic imaging technique that enables visualization of the fine-grained fiber architecture with high resolution (micrometer scale) (Axer et al. 2011b). It does not require chemical staining, though it does involve complex and computationally intensive and image processing steps and allows for high-resolution analysis of the three-dimensional orientation and microstructure of myelinated fibers. In contrast, traditional myelin staining provides only two-dimensional information, making it difficult to accurately characterize fiber orientation and subtle structural changes. Compared with diffusion tensor imaging (millimeter scale), it also exhibits advantages in resolving cross fibers (Zeineh et al. 2017) and capturing the distribution of cell bodies to identify hippocampal subfields. However, improvements are still necessary in the image registration process for the accurate 3D reconstruction of these 2D high resolution images, since the perfect inter-section alignment of individual fibers remains problematic, and thus limits the use of PLI data for tract tracing purposes.

Mapping human hippocampal connectivity is essential for understanding its role in normal memory functions and its dysfunctions in neurodegenerative disorders such as Alzheimer’s disease (Zeineh et al. 2017). However, our knowledge of human hippocampal circuitry is largely inferred from tracer studies conducted in animals such as rats and monkeys (Chrobak and Amaral 2007; Kondo et al. 2009). To overcome this limitation, Zeineh et al. (2017) used high-resolution PLI images to directly dissect and compare hippocampal connectivity in three human and two vervet monkey hemispheres. They have clearly identified multiple components of the perforant path system in hippocampal complex, which includes (1) superficial fiber sheets starting from the entorhinal cortex that project to the presubiculum and parasubiculum; (2) intermixed transverse and longitudinal angular bundle fibers perforating the subiculum before projecting to the CA fields and molecular layer of the DG; and (3) a prominent alvear pathway extending from the angular bundle to the CA fields. Moreover, they provided powerful evidence for the existence of the endfolial path in the vervet brain, a feature previously observed in humans (Lim et al. 1997).

Since its development in the early 1990s (Bandettini et al. 1992; Belliveau et al. 1991; Ogawa et al. 1990, 1992), MRI has become an established non-invasive in vivo method enabling longitudinal studies aiming to understand the impact of aging on the brain’s structural organization. More importantly, since is also widely used in clinical settings, it bridges the gap between basic neuroscience and clinical applications, thus facilitating translational neuroscience. The location and convoluted nature of the hippocampus make it a difficult brain region to study with conventional MRI, and in vivo MRI efforts have gone hand in hand with the development of protocols for labeling hippocampal regions with the aid of ultra-high resolution ex vivo MRI datasets (Augustinack et al. 2010; Beaujoin et al. 2018; McCrea et al. 2025; Modo et al. 2023). Importantly, some of these studies combined ex vivo MRI analysis with subsequent histologic processing of the scanned tissue, thus providing cytoarchitectonic validation of their MRI parcellation criteria (Augustinack et al. 2010, 2014, 2013; González Fuentes et al. 2023). Advances in MRI technology have reduced the severity of partial volume artifacts to which the hippocampus is prone, resulting in an increasing body of literature aiming to characterize the in vivo structural properties of hippocampal regions as well as their distinct functional roles. Despite these improvements, hippocampal regions and layers remain difficult to identify in vivo, as evidenced by differences in the existing manual or automated segmentation protocols. Yushkevich et al. (2015a) performed a quantitative comparison of 21 protocols used by the in vivo imaging community to create a segmentation protocol integrating the anatomical landmarks and image intensity cues most frequently used to delineate hippocampal regions. The ensuing boundary dispersion maps with hippocampal regions and layers were provided as supplementary material accompanying the harmonized protocol (Yushkevich et al. 2015a), and integrated into automated hippocampal segmentation tools (e.g., MAGeT-Brain, Pipitone et al. 2014; Yushkevich et al. 2015b) to facilitate their widespread use in future basic and clinical neuroscience approaches.

Diffusion MRI (dMRI) is the most common non-invasive method for the in vivo reconstruction, visualization and analysis of white matter tracts in the brain. Despite the enormous progress made in recent years, dMRI remains generally susceptible to partial volume effects, eddy currents, and magnetic field inhomogeneities (Assaf et al. 2019; Behrens et al. 2014; Karat et al. 2024; Mori and Zhang 2006; Van Essen et al. 2014). Furthermore, accuracy of current modeling and tractography approaches is limited by complex fiber geometries and becomes even less reliable in highly convoluted brain regions. In addition, these important methodological drawbacks are exacerbated by the proximity of the hippocampus to the lateral ventricle and by the interleaved C-shaped configuration of its FD and CA regions. Diffusion tensor imaging analysis of ex vivo high resolution structural dMRI data enables visualization of the complex trajectory of the perforant path, though not of intra-hippocampal circuitry (Augustinack et al. 2010; Beaujoin et al. 2018; Coras et al. 2014; Zeineh et al. 2012). Several components of the Papez circuit, including the perforant path and fornix, though not the mossy fibers or the Schaffer collaterals could also be visualized in vivo using 7-Tesla super-resolution MRI and track-density imaging with a seed-based tracking analysis (Choi et al. 2019).

Therefore, further improvements at both the hardware and software levels are necessary before MRI can be considered a dependable method for the in vivo analysis of the microstructural organization of the hippocampus, although recent advancements suggest promising progress in this direction. Boulant et al. (2024) successfully acquired in vivo human brain images at 11.7 T, achieving mesoscale resolutions with short acquisition times while maintaining a high signal-to-noise and contrast-to-noise ratio. Even higher field strength (e.g., 14 T) MRI systems are available for small animals, and are also planned for the scanning of human brains (Budé et al. 2025; Hike et al. 2025). The widespread use of these ultra-high-field MRI systems will enable more detailed brain imaging, leading to a better understanding of the relationship between hippocampal structure and function organization principles, and providing new insights into disease mechanisms.

## Outlook

The high-dimensional nature of many modern datasets together with the ever-increasing amount of data made publicly available by large-scale collaborative initiatives have prompted the introduction of artificial intelligence in neuroscience (Amunts et al. 2022). Deep learning and training data curated by neuroanatomists were used to automatically segment cortical layers throughout the entire BigBrain (Amunts et al. 2013), a 3D volumetric reconstruction of a postmortem human brain processed for the visualization of cell bodies (Wagstyl et al. 2020). The ensuing segmentations were verified by expert anatomists and constitute first quantitative 3D laminar atlas of the entire human cerebral cortex (Wagstyl et al. 2020). A convolutional neural network was also used to enable the automated mapping of cytoarchitectonically identified areas in a large number of sections through a human brain based on annotations of a target area in only two training sections (Schiffer et al. 2021). Again, the annotations and the validation were performed by neuroanatomists (Schiffer et al. 2021). Recently, Oberstrass et al. (2024) combined a geometric unfolding method with deep texture features extracted from 3D-PLI data (Axer et al. 2011a) using self-supervised contrastive learning to analyze the regional organization of the human hippocampus. It must be noted that HippUnfold, the pipeline used for the geometric unfolding, only samples a subset of CA layers (DeKraker et al. 2023; Karat et al. 2023), and thus only captures the complexity of the pyramidal and oriens layers. However, the hippocampal subfields highlighted by this approach align with classical divisions as identified by a neuroanatomist, thus demonstrating that PLI and this analytical framework can be effectively used to study the regional organization of hippocampal microcircuitry (Oberstrass et al. 2024).

Beneath a superficially conserved framework, the hippocampus has undergone evolutionary changes in aspects as diverse as subfield expansion, enhanced synaptic plasticity mechanisms, or connectivity patterns. Cross-species analyses can significantly advance our understanding of causal relationships by shedding light on structural changes associated with the need to adapt to diverse ecological and functional requirements. Comparative approaches also enhance the translational value of animal models by ensuring that findings more accurately reflect human hippocampal organization and function, ultimately accelerating the development of targeted therapies for neurologic and psychiatric disorders. Existing methods offer distinct advantages in uncovering hippocampal features across species, spanning multiple spatial and temporal scales, and generating large-scale, multidimensional datasets. Such datasets urgently require a comprehensive analytical framework such as that provided by the ‘common space approach’ to integrate and explore them across different species (Mars et al. 2021). Although initially devised to overcome methodological restrictions caused by morphologic and anatomical variations across different species, the ‘common space approach’ proposed by Mars et al. (2018) also serves to perform vertical translation analyses through the integration of multiple modalities via, e.g., a ‘connectivity space’ or ‘gene space’, thus enabling simultaneous analysis of different aspects of brain organization within a given species (Beauchamp et al. 2022; Mars et al. 2021). Application of such a framework to the hippocampus would accelerate the integration of high-resolution anatomical data (e.g., synaptic morphology, cellular distribution patterns), temporally precise data (e.g., results from electrophysiology or fMRI studies), and computational models that can link microcircuit properties to entire region or even whole-brain dynamics and thus facilitate our understanding of the relationship between its structural and functional segregation.

Concluding, future methodological advances in the field of brain research must necessarily be comparative and multidisciplinary in nature, combining the expertise of physicists, computer neuroscientists and classical neuroanatomists.
